# Fullerene-Based Photoactive Layers for Heterojunction Solar Cells: Structure, Absorption Spectra and Charge Transfer Process

**DOI:** 10.3390/ma8010042

**Published:** 2014-12-25

**Authors:** Yuanzuo Li, Dawei Qi, Peng Song, Fengcai Ma

**Affiliations:** 1 College of Science, Northeast Forestry University, Harbin 150040, Heilongjiang, China; E-Mail: qidw9806@126.com; 2 Department of Physics, Liaoning University, Shenyang 110036, Liaoning, China; E-Mail: mafengcai@lnu.edu.cn

**Keywords:** heterojunction solar cells, fullerene derivatives, polymer (APFO_3_), absorption spectra, charge transfer

## Abstract

The electronic structure and optical absorption spectra of polymer APFO_3_, [70]PCBM/APFO_3_ and [60]PCBM/APFO_3_, were studied with density functional theory (DFT), and the vertical excitation energies were calculated within the framework of the time-dependent DFT (TD-DFT). Visualized charge difference density analysis can be used to label the charge density redistribution for individual fullerene and fullerene/polymer complexes. The results of current work indicate that there is a difference between [60]PCBM and [70]PCBM, and a new charge transfer process is observed. Meanwhile, for the fullerene/polymer complex, all calculations of the twenty excited states were analyzed to reveal all possible charge transfer processes in depth. We also estimated the electronic coupling matrix, reorganization and Gibbs free energy to further calculate the rates of the charge transfer and the recombination. Our results give a clear picture of the structure, absorption spectra, charge transfer (CT) process and its influencing factors, and provide a theoretical guideline for designing further photoactive layers of solar cells.

## 1. Introduction

Organic heterojunction photovoltaic devices have received increasing scientific attention owing to their flexibility, ease of processing, potentially low cost and the long-term sustainability advantages of organics [1,2,3,4], as well as being viewed as promising alternatives for established silicon based systems. For a heterojunction solar cell, the active layer is sandwiched between a transparent indium tin oxide (ITO) anode and a low-work-function metal cathode, comprised of a conjugated polymer as donor (D) and a fullerene derivative as acceptor (A). During photo-excitation, firstly the active layer absorbs the solar photons to create excitons, followed by dissociating into free holes and electrons in the D/A interface; secondly, holes and electrons move through the donor and acceptor channels to anodes and cathodes, respectively; subsequently, charges are collected at the electrodes, resulting in the generation of electrical power. Therefore, to construct an efficient artificial photoactive layer, the following characteristics [5,6] are required: (a) the capture of absorption light obtained by antenna molecules; and (b) the absorption of light must lead to direct electron transfer from D to A; and (c) the charge transfer rate must be larger than the charge recombination rate. Usually, the candidates of an electron transfer system with high-efficiency are covalently linked donor and acceptor moieties; for example, some photosensitizing electron donors such as porphyrin, phthalocyanine and ruthenium phthalocyanine, were covalently linked to fullerene [7,8,9]. Another approach is a mixture of fullerene with an electron donor, such as poly(3-hexylthiophene) (P3HT): [60]PCBM, Poly(p-phenylene vinylene) (PPV): [60]PCBM, metallophthalocyanine: fullerene [10,11,12,13,14],* etc*.

As an important assisting method, theoretical simulation may provide the clue to understand the microscopic mechanism behind the experimental phenomenon. Theodorakopoulos* et al.* [15] investigated the electronic structure and in particular the effect on the chemical properties of the pyrrolidine nitrogen atom of fullerene as well as of additional substituted groups with density functional theory (DFT) and time-dependent DFT (TD-DFT) methods. The geometries, electronic structures, polarizabilities and hyperpolarizabilities, and UV-vis spectra of metallo phthalocyanine dyes and metallophthalocyanine-fullerene supramolecules were studied [16]. The relationship between charge density and mobility of fullerenes was revealed by using first-principles calculation [17]. Barszcza* et al.* [18] reported a theoretical investigation on the electronic absorption spectra of fullerene-thiophene-derived dyads, and they found the strongest excitations in the dyads are mainly related to the excitations of the fullerene part with some influence from the thiophene-derived part and intramolecular charge transfer processes. Our previous research also demonstrated that some states are intramolecular charge transfer states, and others belong to locally excited states, and predicted that electron transfer for the intramolecular charge transfer state takes place more easily, according to the calculated results of the electronic coupling matrix elements [19,20].

Though much attention has been paid to the utility of C60 derivatives as an artificial photoelectric conversion system, little work has been done on the light absorption characteristics and the charge transfer process of C70 derivatives, especially for the individual C70 derivatives and the mixture of C70 derivatives with counter donor. The effect of C60 and C70 on the optical response and efficiency of interfacial charge transfer still needs to be studied in detail. In the current work, we performed calculations of density functional theory (DFT) and time-dependent density functional theory (TDDFT) to obtain the geometric, electronic structures and the absorption spectra of the mixed fullerene/polymer complex, that is, [70]PCBM/APFO_3_ ([6,6]phenyl-C71-butyric acid-methyl ester) and [60]PCBM/APFO_3_ on the basis of experimental report [21]; the name APFO_3_ is the abbreviation of APFO_3_ (poly[2,7-(9,9-dioctylfluorene)-*alt*-5,5-(4,7′-di-2-thienyl-2′,1′,-3-benzothiadiazole)]. The parameters affecting charge transfer and charge recombination, were estimated and compared. Moreover, the developed 3D real-space analysis was used to investigate the excited states feature and charge transfer properties of the binary system.

## 2. Methods

**Figure 1:**
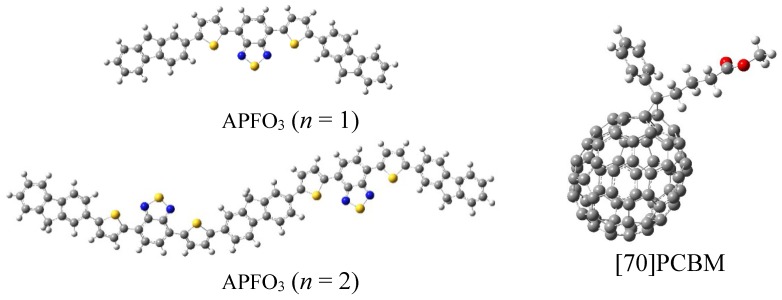
Structures of APFO_3_ (poly[2,7-(9,9-dioctylfluorene)-*alt*-5,5-(4,7′-di-2-thienyl- 2′,1′,-3-benzothiadiazole)] (*n* = 1 and *n* = 2) and C70-fullerene based acceptor.

All the quantum chemical calculations were done with Gaussian 09 suite [22]. The molecular structures of APFO_3_, [70]PCBM/APFO_3_ and [60]PCBM/APFO_3_ can be seen from Figure 1. The side chains of APFO_3_ were replaced by hydrogen atoms in order to save computational cost, on consideration that they merely aid in improving solubility and have negligible influence on optical properties [23,24]. Although the omission of the side chains is a common decision in this field, it should be done with caution because the side chains can affect conformational torsion of the backbone of some oligomers [25]. The ground state geometries were optimized with density functional theory (DFT) [26], using B3LYP functional [27,28,29] and 6-31G (D) basis set. For the calculations of inner reorganization energies, the cationic ground state geometry of APFO_3_, and anionic ground state geometries of [70]PCBM and [60]PCBM were optimized, using the DFT//B3LYP/6-31G(D). Then the energies of neutral acceptors at the anionic geometry and the optimal ground-state geometry were calculated by using the DFT//B3LYP/6-31G(D), respectively; and the energies of the radical cation at the neutral geometry and optimal cation geometry were calculated on the same functional and basis set. Based on the optimized neutral structures, the time-dependent DFT (TD-DFT) method [30] with long-range corrected functional Cam-B3LYP [31] and basis set 6-31G (D) was used to obtain the optical absorption properties. To calculate the charge transfer integral (electronic coupling matrix), the *Generalized Mulliken*-*Hush* (GMH) model and the finite field method on the excitation energy of the donor-acceptor heterojunction were employed (which will be discussed below).

To visualize charge transfer on their electronic transitions, three dimensional (3D) cube representations were used, and 3D charge difference density indicated that the electronic redistribution involving the whole structure takes place upon excitation [32,33,34,35]. The charge difference density is defined as:

(1) $$\Delta {\text{ρ}}_{uu}(r)=\sum_{\begin{array}{l} a\in unocc\\ i,j\in occ \end{array}}C_{uaj}C_{uai}\phi_{j}(r)\phi_{i}(r)-\sum_{\begin{array}{l} a,b\in unocc\\ i\in occ \end{array}}C_{ubi}C_{uai}\phi_{b}(r)\phi_{a}(r)$$

where $C_{uai}$ is the *u*th eigenvector of the single configuration interaction (CI) Hamiltonian on the basis of the occupied Hartree-Fock molecular orbital ϕ *_i_*(*r*) and the unoccupied ϕ *_a_*(*r*) orbital [32,33]; in this equation the first and the second terms stand for hole and electron, respectively.

## 3. Results and Discussion

### 3.1. Energy Levels and Band Gap

**Figure 2:**
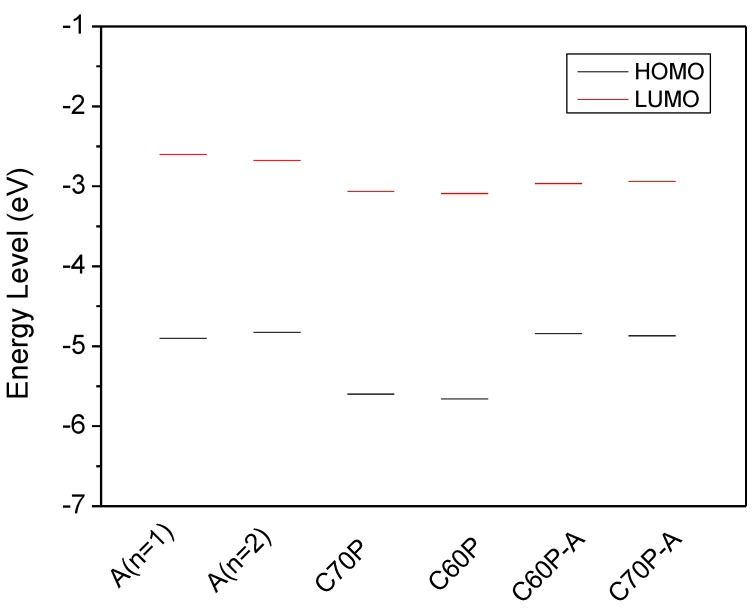
Energy levels of polymers and fullerene, where (APFO_3_)_n=1_, (APFO_3_)_n=2_, [C70]PCBM, [C60]PCBM, [C60]PCBM/(APFO_3_)_n=1_ and [C70]PCBM/(APFO_3_)_n=1_ are abbreviated as A(*n* = 1), A(*n* = 2), C70P, C60P, C60P-A and C70P-A, respectively.

The calculated energies of the highest occupied orbital (HOMO) and the lowest unoccupied orbital (LUMO) are shown in Figure 2, and the detailed results are listed in Table S1. As shown in Figure 2, the differences of energy levels between HOMO and LUMO for polymer APF03 are small for different units (*n* = 1 and *n* = 2), and the band gap is calculated to be 2.274 eV and 2.151 eV for *n* = 1 and *n* = 2, respectively; the calculated result of unit *n* = 1 agrees well with the experimental result (2.2 eV) [21]. As another binary system, the energy levels of C70 and C60 derivatives have the diversity to reduce in comparison with APFO_3_. The LUMO of C70P is slightly higher than that of C60P. While, the LUMOs of the binary system are closed to that of fullerenes, their HOMOs verge on HOMOs of APFO_3_, which leads to charge transfer controlling by transition from HOMO to LUMO and can take place from APFO_3_ to fullerenes. Compared to the isolated donor or acceptor, the donor-acceptor complex has a decreased trend of HOMO-LUMO band gap.

### 3.2. Optical Absorption of Donor, Acceptor and the Donor-Acceptor Complex

**Figure 3:**
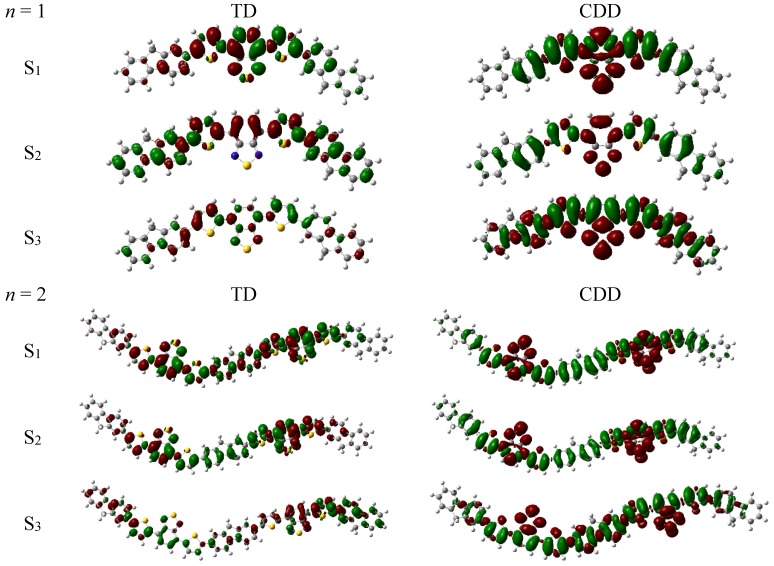
Transition density (TD) and charge difference density (CDD) of polymer (*n* = 1 and *n* = 2).

Based on optimized ground-state structure of APFO_3_, vertical excitation energies and oscillator strengths for the five excited states were calculated, which are listed in Table 1. For *n* = 1 and *n* = 2, the absorption spectra cover the UV-visible region, and have one common property, * i.e.*, their first excited state (S_1_) has high oscillator strength, compared to the other energetically low lying states. Transition density in Figure 3 shows the strength and orientation of the transition moment for calculated excited states. For *n* = 1, red electrons are mainly located on the left unit and green holes reside on the right unit, and thus the transition moment is singlet direction. In comparison, the orientation of the transition moment for *n* = 2 is unchanged, and the electron and hole are distributed over two monomers, which results in the increased strength of the transition moment. Due to the proportional relationship between oscillator strength with the transition energy (*E_ge_*) and transition moment (μ*_ge_*), $f=(8{\text{π}}^{2}m_{e}/3{\text{e}}^{2}h)E_{ge}{\text{μ}}_{ge}^{2}$ [36,37], APFO_3_ (*n* = 2) displays a larger oscillator strength than APFO_3_ (*n* = 1) under the condition of similar transition energy (Table 1). The week absorption of S_2_ can be explained by TD analysis, and Figure 3 shows there are the two sub-transition dipole moments with the “tail to tail” character since more holes are mainly localized on both sides of APFO_3_, which results to a large extent in the weakness of the total transition dipole moment. So the total transition dipole moment of S_2_ state is smaller than that of S_1_ state. Turning to the charge transfer character of APFO_3_, the redistribution of electron density during photo-excitation was visualized with charge difference density (see Figure 3). It was found that S_1_ and S_2_ have some intramolecular CT character, where electron transfer is transferred from two-sided fluorene and thiophene units to the middle unit; while the S_3_ state at 3.78 eV is essentially an π−π* excited state.

**Table 1:** Calculated transition energies (eV, nm) and oscillator strengths (*f*) for polymer (*n* = 1 and *n* = 2).

States	*n* = 1		*n* = 2		Experiment
	eV (nm)	*f*	eV (nm)	*f*	nm
S_1_	2.48(500.84)	1.3006	2.40(515.68)	2.8379	540
S_2_	3.51(353.23)	0.0299	2.52(491.41)	0.0625	–
S_3_	3.78(328.04)	1.3606	3.40(364.33)	0.1901	384
S_4_	4.09(302.68)	0.0862	3.49(355.25)	0.0006	–
S_5_	4.31(287.55)	0.0023	3.67(337.96)	1.7567	–

**Figure 4:**
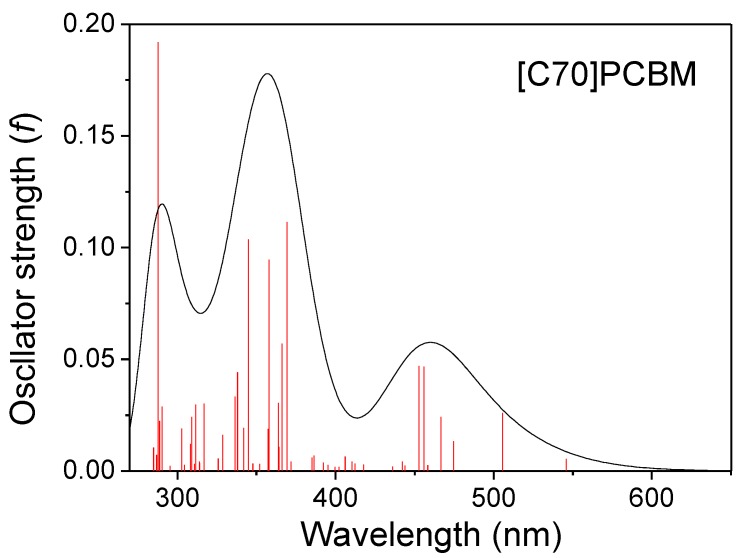
Absorption spectra of [C70]PCBM.

**Figure 5:**
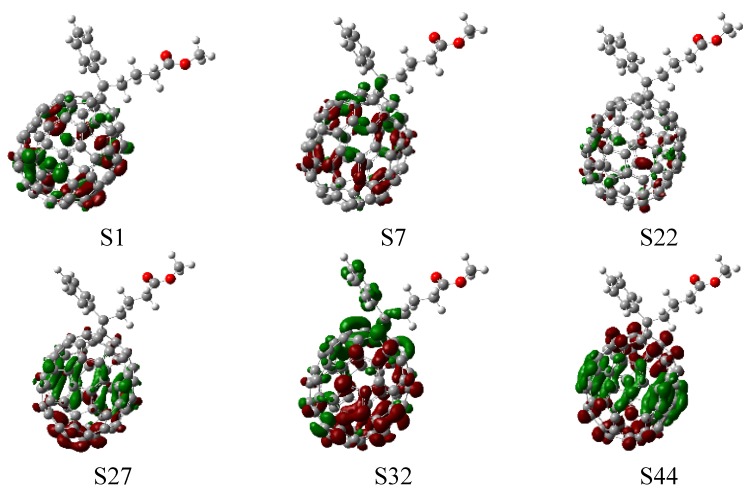
Charge difference density (CDD) of [C70]PCBM, where the green and red stand for the hole and electron, respectively.

UV-visible spectra of [70]PCBM were simulated on the basis of the calculated fifty excited states (see Table S2), and transition energies and oscillator strengths were interpolated by a Gaussian convolution with the full width at half-maximum of 0.4 eV. As shown in Figure 4, the simulated UV-visible absorption spectra of [70]PCBM exhibits three broad and dense bands. The first absorption band of [70]PCBM is at about 450 nm, and is mainly composed of two bright states (S7 and S6), which come from a strong local excitation of C70 because photon-induced distribution of electron-hole pairs only locates on C70 (see CDD in Figure 5, where several typical excited states are listed; more excited states can be seen in Figure S1). For the other two bands, the dominated higher singlet states (S22, S26, S30, S33 and S48) have local excitation character to some extent. Note that, there are typical charge redistributions for the C70 derivative, that is, one is charge transfer from the middle body to the bottom part (S27 state); the other is the stronger intramolecular CT from the top benzene of the C70 derivative to the bottom part (S32 state); the final kind of CT only occurs on inner C70 from the middle body of C70 to both sides of the upper and lower (S44 state).

**Figure 6:**
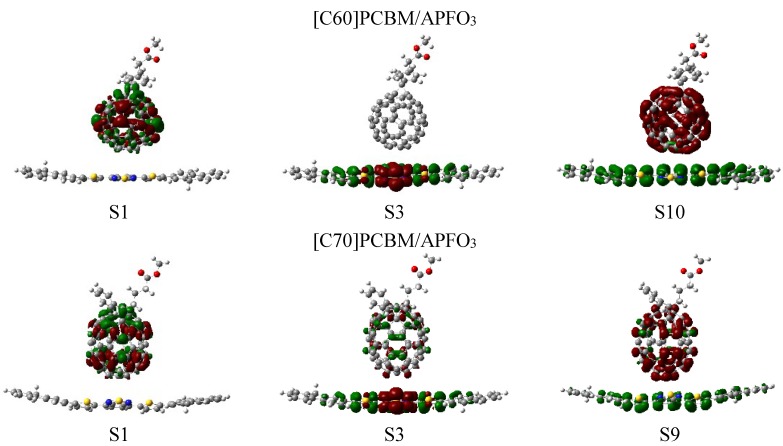
Charge difference density (CDD) of [C60]PCBM/APFO_3_ and [C70]PCBM/APFO_3_, where the green and red stand for the hole and electron, respectively.

The charge difference densities of [C60]PCBM/APFO_3_ and [C70]PCBM/APFO_3_ are shown in Figure 6, and transition energies and oscillator strengths are listed in Table 2. For [C60]PCBM/APFO_3_, its excited states are classed as three kinds of excitation, in which S1 and S3 states represent two typical locally excited states. Table 2 shows that the strongest absorption peak of [C60]PCBM/APFO_3_ corresponding to S3 state with *f* = 1.1259, and electron-hole pairs is located on APFO_3_ (for S3). This state is a local-excited state; however, intramolecular charge transfer takes places on the molecular skeleton of APFO_3_, which displays the same character as the CT states of APFO_3_ monomer. The S1, S2, S4–S9, S11, S13, S14, S15, S17–S20 states are local-excited states by exciting C60 (See Figure S2). Additionally the lowest intermolecular charge transfer excited state is the S10 state, peaking at 433 nm (Figure 6); this state can be expected to undergo a direct electron transfer from donor to acceptor, resulting in the charge separation. Similar CT excited states are found to be S12 and S16 states (See Figure S2).

**Table 2:** Calculated transition energies (eV, nm) and oscillator strengths (*f*) for [C60]PCBM/APFO_3_ and [C70]PCBM/APFO_3_, respectively.

States	[C60]PCBM & APFO_3_		[C70]PCBM& APFO_3_	
	eV (nm)	*f*	eV (nm)	*f*
S_1_	2.42(511.43)	0.0017	2.27(545.29)	0.0014
S_2_	2.46(504.84)	0.0026	2.45(506.59)	0.1925
S_3_	2.48(500.52)	1.1259	2.48(500.88)	0.9239
S_4_	2.53(490.32)	0.0004	2.61(474.33)	0.0127
S_5_	2.55(486.25)	0.0000	2.66(466.35)	0.0159
S_6_	2.68(463.09)	0.0001	2.71(457.64)	0.0006
S_7_	2.73(454.88)	0.0004	2.72(456.05)	0.0432
S_8_	2.78(445.36)	0.0000	2.74(452.86)	0.0663
S_9_	2.84(437.07)	0.0003	2.79(443.97)	0.0020
S_10_	2.86(433.12)	0.0053	2.79(443.84)	0.0024
S_11_	2.87(431.69)	0.0008	2.81(442.00)	0.0023
S_12_	2.94(421.83)	0.0005	2.85(435.34)	0.0000
S_13_	2.95(419.91)	0.0013	2.89(428.27)	0.0045
S_14_	2.99(415.21)	0.0018	2.96(419.22)	0.0006
S_15_	3.00(412.93)	0.0001	2.97(416.85)	0.0009
S_16_	3.09(401.58)	0.0005	3.01(411.92)	0.0022
S_17_	3.10(400.60)	0.0002	3.02(410.38)	0.0020
S_18_	3.14(394.37)	0.0010	3.05(405.93)	0.0050
S_19_	3.18(389.81)	0.0153	3.08(402.16)	0.0000
S_20_	3.46(358.09)	0.0029	3.10(399.78)	0.0000

For [70]PCBM/APFO_3_, the charge difference density in Figure 6 reveals that there are also three kinds of excited state: (a) local-excited state of C70 (S1, S4, S5–S8, S11, S12, S15–S20, see Figure S3); (b) an entire intermolecular CT state (S9, S10, S13, S14) and (c) an intramolecular CT state of APFO_3_coupled with local-excited states of [C70]PCBM (S2 and S3); the lowest intermolecular charge transfer excited state is the state S9, peaking at 444 nm.

### 3.3. Rate of Charge Transfer in the Marcus Theory

The rates of exciton dissociation and charge recombination were evaluated by the Marcus theory [38]:

(2) $$k=\sqrt{\frac{4{\text{π}}^{3}}{{\text{h}}^{2}\text{λ}k_{B}T}}{\left|V_{DA}\right|}^{2}\exp\left(-\frac{{\left(\Delta G+\text{λ}\right)}^{2}}{4\text{λ}k_{B}T}\right)$$

where λ is the reorganization energy, *V_DA_* is the electronic coupling (charge-transfer integral) between donor and acceptor, Δ*G* is the free energy change for the electron transfer reaction, *k_B_* is the Boltzmann constant, h is Planck’s constant, and* T* is the temperature (we set *T =* 300K in our calculations). Firstly, the *Generalized Mulliken*-*Hush* (GMH) model was used to estimate the charge transfer integral (electronic coupling matrix) [39]. In terms of the two states (*S*_0_ and *S*_n_ states) the formulation, electronic coupling matrix can be written as:

(3) $$V_{DA}=\frac{{\text{μ}}_{tr}\Delta E}{\sqrt{{\left(\Delta \text{μ}\right)}^{2}+4{\left({\text{μ}}_{tr}\right)}^{2}}}$$

This expression involves the energy difference Δ*E* and transition dipole moment μ*_tr_* as well as the corresponding dipole moment difference Δμ between the initial and final electronic states. The Δμ in the above equation was calculated using the Hellmanne Feynman theorem, as the analytical derivative of the excited-state energy with respect to an applied electric field. For the dimer system of fullerene/polymer, the first charge transfer state for [70]PCBM/APFO_3_ and [C60]PCBM/APFO_3_ corresponding to the pure intermolecular charge transfer excited state identified as the fully charged separation state, pointed to the final state in order to obtain the electronic coupling. The transition energy dependent on the static electric field F can be expressed as [40]:

(4) $$E_{exc}(F)=E_{exc}(0)-\Delta \text{μ}F-\frac{1}{2}\Delta \text{α}F^{2}$$

where $E_{exc}(0)=\Delta E$ is the excitation energy at zero field, Δα is the change in the polarizability. For the charge transfer state, Table 3 shows the fitted values of Δμ for [C60]PCBM/APFO_3_ and [C70]PCBM/APFO_3_ (13.39286 a.u. and 10.41667 a.u.), respectively. According to Equation (3), the electronic coupling strengths (*V_DA_*) are calculated to be 329.2 cm^−1^ (0.04081 eV) and 260.2 cm^−1^ (0.03226 eV), respectively.

**Table 3:** Calculated dipole moment of state-to-state and coupling strength.

Complex	States	Δ*U* (a.u.)	*U* (a.u.)	*V_DA_* (cm^−1^)
[C60]PCBM/APFO_3_	S_10_	13.39286	0.1910	329.2
[C70]PCBM/APFO_3_	S_9_	10.41667	0.1204	260.2

In the exciton dissociation and charge recombination, $\Delta G=\Delta G_{CT}$ and $\Delta G_{CR}$, respectively. The $\Delta G_{CR}$ can be estimated with [41]:

(5) $$\Delta G_{CR}=E_{IP}(D)-E_{EA}(A)$$

where $E_{IP}(D)$ and $E_{EA}(A)$ are the ionization potential of the donor and electron affinity of the acceptor, respectively. These quantities are normally estimated from the energies of the highest occupied molecular orbital and lowest unoccupied molecular orbital of the donor and acceptor [41] (see Table S1), respectively. The calculated Δ*G_CR_* are −1.81 eV for [C60]PCBM/APFO_3_ and −1.837 eV for [C70]PCBM/APFO_3_, as can be seen from Table 4, and negative values signify the process of electron recovery is spontaneous thermodynamically for these two systems. Δ*G_CT_* can be estimated by using the Rehm-Weller equation, $\Delta G_{CT}=-\Delta G_{CR}-\Delta E_{0-0}$, where $\Delta E_{0-0}$ is the energy of the lowest excited state of free-base donor. The calculated Gibbs Free energy differences Δ*G_CT_*, are all negative values (see Table 4), which means that electron transfer is thermodynamically favorable for these two systems. There is a directly competitive process between intermoleular charge transfer and charge recombination, and thus it is expected to maximize intermoleular charge transfer and minimize charge recombination for designing high-efficiency solar cells.

**Table 4:** Dynamic parameters for [C60]PCBM/APFO_3_ and [C70]PCBM/APFO_3_.

Complex	Δ *G_CR_*	λ	Δ *G_CT_*	*V*_DA_	*K*_CT_ (×10^13^)	*K*_CR_ (×10^7^)
[C60]PCBM/APFO_3_	−1.810	0.7	−0.6655	0.04082	3.2811	0.13517
[C70]PCBM/APFO_3_	−1.837	0.7	−0.6400	0.03265	2.0304	0.036515

Furthermore, to calculate reorganization energy λ, we optimized the charged APFO_3_ and fullerene derivatives, since the inner reorganization energy arises from the change in equilibrium geometry of the donor (D) and acceptor (A) sites consecutive to the gain or loss of electronic charge upon electron transfer. For the outer reorganization energy, it originates from the electronic and nuclear polarization/relaxation of the surrounding medium, which is not easy to estimate quantitatively in the solid state. So, the total reorganization energy in the calculations is adopted from experimental results. The energies of the neutral acceptor (A) at the anionic geometry and optimal ground-state geometry ($E(A^{-})$ and E(A), and subsequently the energies of the cation donor at the neutral geometry and optimal cation geometry (E(D) and $E(D^{+})$) were calculated only individually.

Table 4 shows the calculated inner reorganization energy, electronic coupling and Gibbs free energy difference. The charge transfer (*K_CT_*) and recombination rates (*K_CR_*) can be simulated from these parameters using Equation (2). When comparing APFO_3_/[C60]PCBM with APFO_3_/[C70]PCBM, it was found that the introduction of [C70]PCBM did not obviously increase the value of *K_CT_* (*K_CT_* = 3.2811×10^13^ s^−1^for [C60]PCBM/APFO_3_ and 2.0304 × 10^13^ s^−1^ for [C70]PCBM/APFO_3_) owing to the similar values of λ, Δ*G_CT_* and *V_DA_*. However, it obviously reduces the rate of charge recombination, and the value of *K_CR_* is calculated to be 0.13517 × 10^7^ s^−1^ (for [C60]PCBM/APFO_3_) and 0.036515 × 10^7^ s^−1^ (for [C70]PCBM/APFO_3_), respectively.

### 3.4. Effect of Electronic Field on CT Rate

The estimation of electronic field effect on the rate of CT requires information of the external electronic field dependent *V_DA_* and Δ*G*. When considering this kind of perturbation, the electronic field has influence on the free energy by means of additional change energy Δμ*F* (where μ and *F* represent the dipole moment of a radical pair and the strength of the external electronic field), and thus under the external electronic field, ΔG(F≠0)=ΔG(F)−ΔuF (where *F* ≠ 0). The external electronic field dependent $V_{DA}(F\neq 0)$ can be induced by extending the GMH model: $V_{DA}(F\neq 0)=\frac{{\text{μ}}_{tr}(F)\Delta E(F)}{\sqrt{{\left(\Delta {\text{μ}}_{F}\right)}^{2}+4{\left({\text{μ}}_{tr}(F)\right)}^{2}}}$. Inserting term of ΔG(F≠0) and $V_{DA}(F\neq 0)$ into Equation (2), we can rewrite the Marcus theory as:

(6) $$\begin{array}{l} k=\sqrt{\frac{4{\text{π}}^{3}}{h^{2}\text{λ}k_{B}T}}{\left|V_{DA}(F\neq 0)\right|}^{2}\exp\left(-\frac{{\left(\Delta G(F=0)-\Delta uF+\text{λ}\right)}^{2}}{4\text{λ}k_{B}T}\right)\\ =\sqrt{\frac{4{\text{π}}^{3}}{h^{2}\text{λ}k_{B}T}}{\left|\frac{{\text{μ}}_{tr}(F)\Delta E(F)}{\sqrt{{\left(\Delta {\text{μ}}_{F}\right)}^{2}+4{\left({\text{μ}}_{tr}(F)\right)}^{2}}}\right|}^{2}\exp\left(-\frac{{\left(\Delta G(F=0)-\Delta uF+\text{λ}\right)}^{2}}{4\text{λ}k_{B}T}\right) \end{array}$$

**Figure 7:**
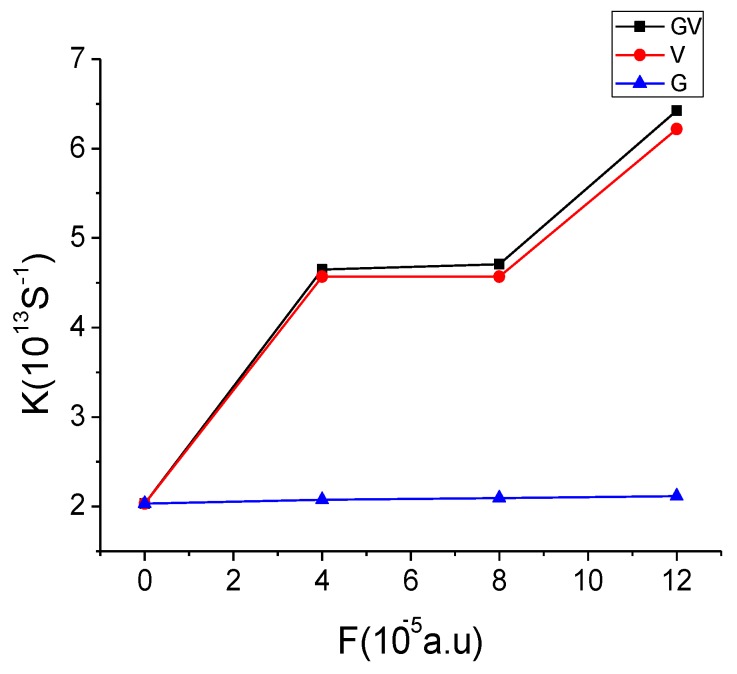
Calculated rates of CT under different electronic fields (a.u.), where blue, red and blank lines are ΔG(F≠0), $V_{DA}(F\neq 0)$ and combination of two factors, respectively.

For estimating $V_{DA}(F\neq 0)$, we calculated the transition energy and transition moments under the varied external electronic field, and fitting Δμ*_F_*, then get the value of *V_DA_* for the S_9_ excited state of [C70]PCBM/APFO_3_ because it is an (intermolecular charge transfer) ICT state. Figure 7 shows the relationship between electronic field and rate of charge transfer. For [C70]PCBM/APFO_3_, it was found that the rate of charge transfer is increased along with the external electronic field as a whole. In addition, we also discussed the effect of the individual values of *V_DA_* and Δ*G* on the rate of charge transfer. When only considering the effect of Δ μF, the rate is almost unchanged with the external electronic field (see blue line in Figure 7),* i.e.*, when *F* = 4 × 10^−5^ a.u., *K_CT_* = 2.0733 × 10^13^ S^−1^, and *F* = 12 × 10^−5^ a.u., *K_CT_* = 2.1141 × 10^13^ S^−1^). When only $V_{DA}(F\neq 0)$ is considered, the CT rate generally grows in response to the increase of the external electronic field. While for *F* = 4 × 10^−5^ and 8 × 10^−5^, the rate is approximately equal, and in a purely computational way the reason can be explained by the fact that the subequal values of transition energies and transition moments result in the very closed $V_{DA}(F\neq 0)$. Noted that, along with the increasing electronic field, obviously the CT rate increases, that is, when *F* = 0, *K_CT_* = 2.0304 × 10^13^ S^−1^ and *F* = 12 × 10^−5^, *K_CT_* = 6.2186 × 10^13^ S^−1^). When the combination of $V_{DA}(F\neq 0)$ and ΔG(F≠0), it was found that the strength and shape by simultaneously considering the two factors are similar with those under the condition of only $V_{DA}(F\neq 0)$, which means that the influence of the electronic coupling matrix on the rate exerts a leading position.

## 4. Conclusions

We have theoretically studied the optical physics characteristics of individual APFO_3_, fullerene, [C60]PCBM/APFO_3_ and [C70]PCBM/APFO_3_. Molecular orbital energies show that the LUMO of [C70]PCBM is slightly higher than that of [C60]PCBM, and the LUMO of the binary system is closed to that of fullerenes. Additionally the HOMOs verge on HOMOs of APFO_3_, which leads to the fact that charge transfer controlled by transition from HOMO to LUMO can take place from APFO_3_ to fullerenes. For the C70 derivative, absorption spectra and charge difference density show that the absorption peak comes from the local excitation of C70 monomer, and there are three kinds of CT originating from intramolecular CT between C70 and the benzene ring and internal composition. Moreover, the excited states of [C60]PCBM/APFO_3_ and [C70]PCBM/APFO_3_ were studied, and locally excited states and charge transfer states were found with CDD analysis. Based on Marcus theory, the calculated rate of charge transfer is of a certain magnitude for [C60]PCBM/APFO_3_ and [C70]PCBM/APFO_3_, while the calculated recombination rate demonstrated the process of charge recombination is more likely to happen for the [C60]PCBM/APFO3 than the [C70]PCBM/APFO_3_. Upon introducing increasing electronic field, the free energy and electronic coupling matrix show a variety of different changes; however, it was found that the changed electronic coupling matrix under increasing electronic field may have even key impacts on the CT rate.

## Supplementary Materials

Supplementary materials can be accessed at: http://www.mdpi.com/1996-1944/8/1/0042/s1.
