# Supplementary material for: Fullerene-Based Photoactive Layers for Heterojunction Solar Cells: Structure, Absorption Spectra and Charge Transfer Process
Source: Materials (Basel). 2014 Dec 25;8(1):42–56. doi: 10.3390/ma8010042 (PMC5455238; doi:10.3390/ma8010042)
Supplement: Supplementary file 1 [file materials-08-00042-s001.pdf]

Supplementary Materials

Table S1. Calculated energies of polymer and fullerenes

Compound	HOMO	LUMO	gap
$(APFO_3)_{n=1}$	−4.9	−2.626	2.274
$(APFO_3)_{n=2}$	−4.826	−2.675	2.151
[C70]PCBM	−5.598	−3.063	2.535
[C60]PCBM	−5.657	−3.09	2.567
[C60]PCBM-$(APFO_3)_{n=1}$	−4.844	−2.966	1.878
[C70]PCBM-$(APFO_3)_{n=1}$	−4.871	−2.939	1.932

Table S2. Calculated transition energies (eV, nm) and oscillator strengths (f) for [70]PCBM.

States	eV(nm)	*f*	States	eV(nm)	*f*
S_1	2.27(545.84)	0.0035	S_{26}	3.46(357.93)	0.0927
S_2	2.45(505.57)	0.0240	S_{27}	3.47(357.27)	0.0170
S_3	2.61(474.65)	0.0113	S_{28}	3.52(352.14)	0.0013
S_4	2.66(466.61)	0.0223	S_{29}	3.56(347.87)	0.0015
S_5	2.70(458.39)	0.0007	S_{30}	3.59(344.97)	0.1018
S_6	2.72(455.90)	0.0448	S_{31}	3.62(342.13)	0.0174
S_7	2.74(452.85)	0.0452	S_{32}	3.67(338.15)	0.0423
S_8	2.79(443.89)	0.0006	S_{33}	3.68(336.59)	0.0313
S_9	2.80(442.08)	0.0024	S_{34}	3.77(328.68)	0.0143
S_{10}	2.84(436.02)	0.0001	S_{35}	3.80(325.89)	0.0037
S_{11}	2.97(417.76)	0.0010	S_{36}	3.91(316.98)	0.0282
S_{12}	3.01(412.32)	0.0015	S_{37}	3.95(314.27)	0.0016
S_{13}	3.02(410.38)	0.0023	S_{38}	3.95(313.98)	0.0022
S_{14}	3.05(406.08)	0.0046	S_{39}	3.98(311.77)	0.0278
S_{15}	3.08(402.29)	0.0000	S_{40}	3.99(310.85)	0.0012
S_{16}	3.10(399.75)	0.0000	S_{41}	4.01(309.30)	0.0223
S_{17}	3.14(395.36)	0.0009	S_{42}	4.02(308.28)	0.0102
S_{18}	3.16(392.34)	0.0018	S_{43}	4.07(304.57)	0.0008
S_{19}	3.21(386.41)	0.0050	S_{44}	4.09(302.89)	0.0171
S_{20}	3.22(385.10)	0.0042	S_{45}	4.19(295.54)	0.0005
S_{21}	3.33(372.02)	0.0024	S_{46}	4.27(290.48)	0.0270
S_{22}	3.36(369.45)	0.1096	S_{47}	4.29(288.95)	0.0205
S_{23}	3.39(366.15)	0.0552	S_{48}	4.31(287.99)	0.1901
S_{24}	3.40(364.37)	0.0089	S_{49}	4.32(287.02)	0.0053
S_{25}	3.41(363.81)	0.0285	S_{50}	4.35(285.13)	0.0085

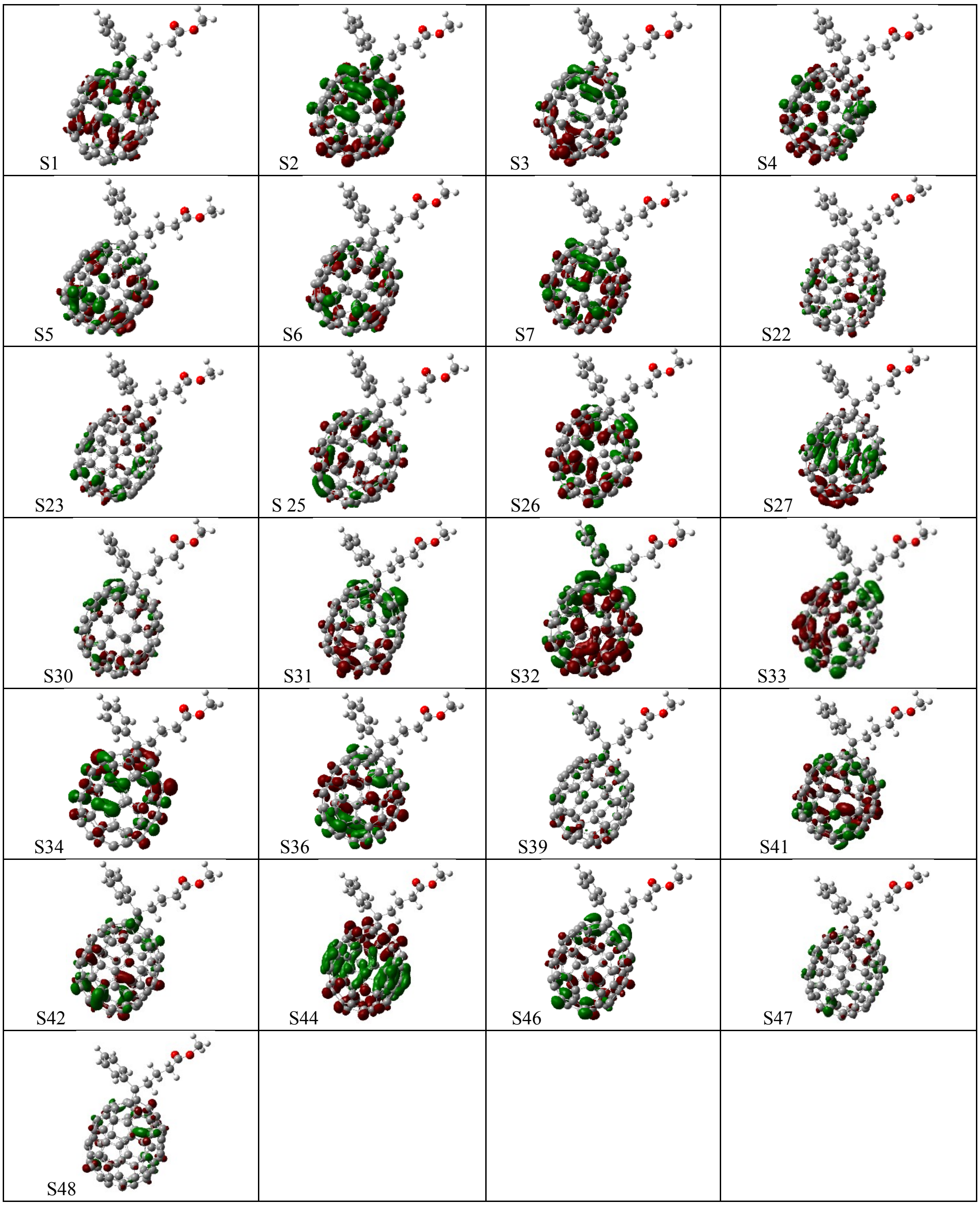

Figure S1. Charge different density (CDD) of [C70]PCBM, where the green and red stand for the hole and electron, respectively.

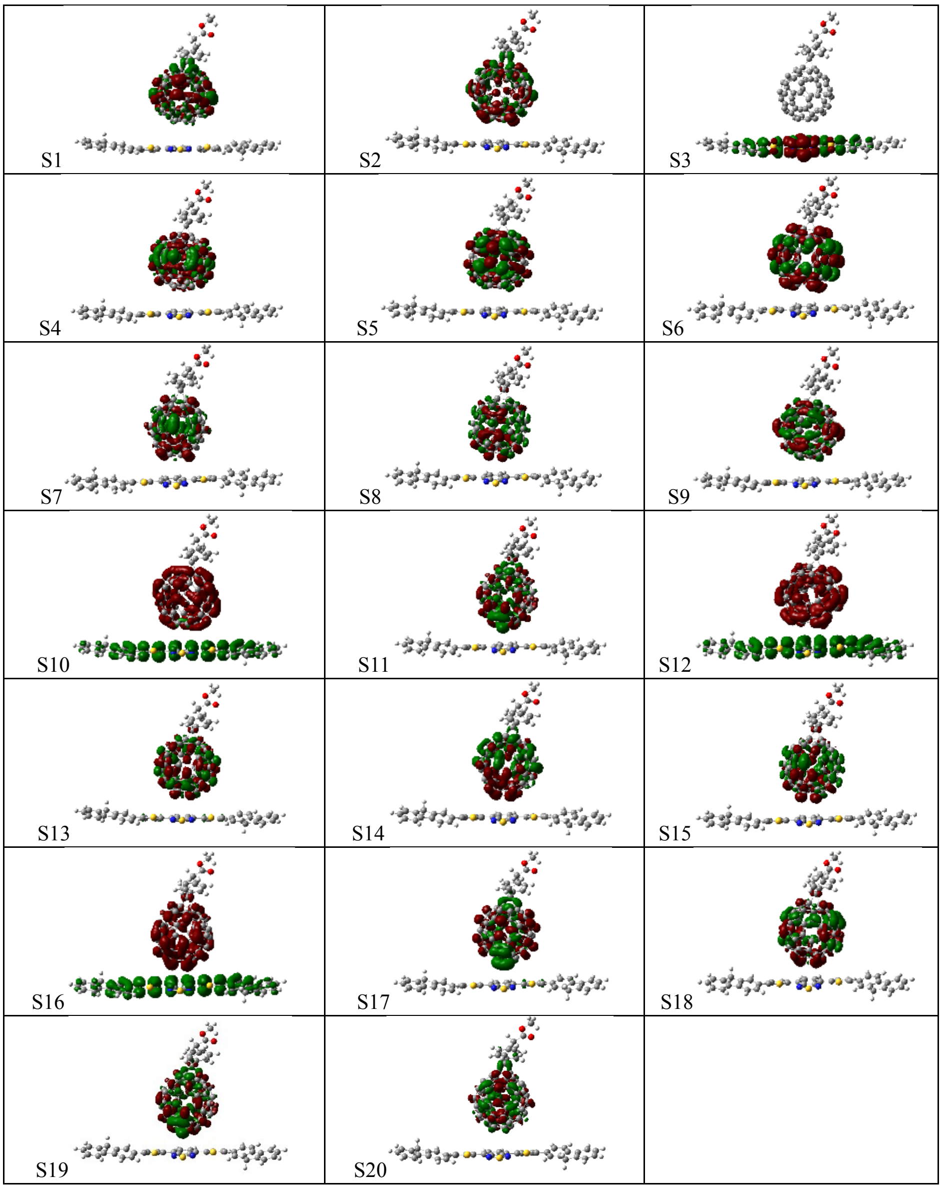

Figure S2. Charge difference density (CDD) of APFO3/[C60] PCBM, where the green and red stand for the hole and electron, respectively.

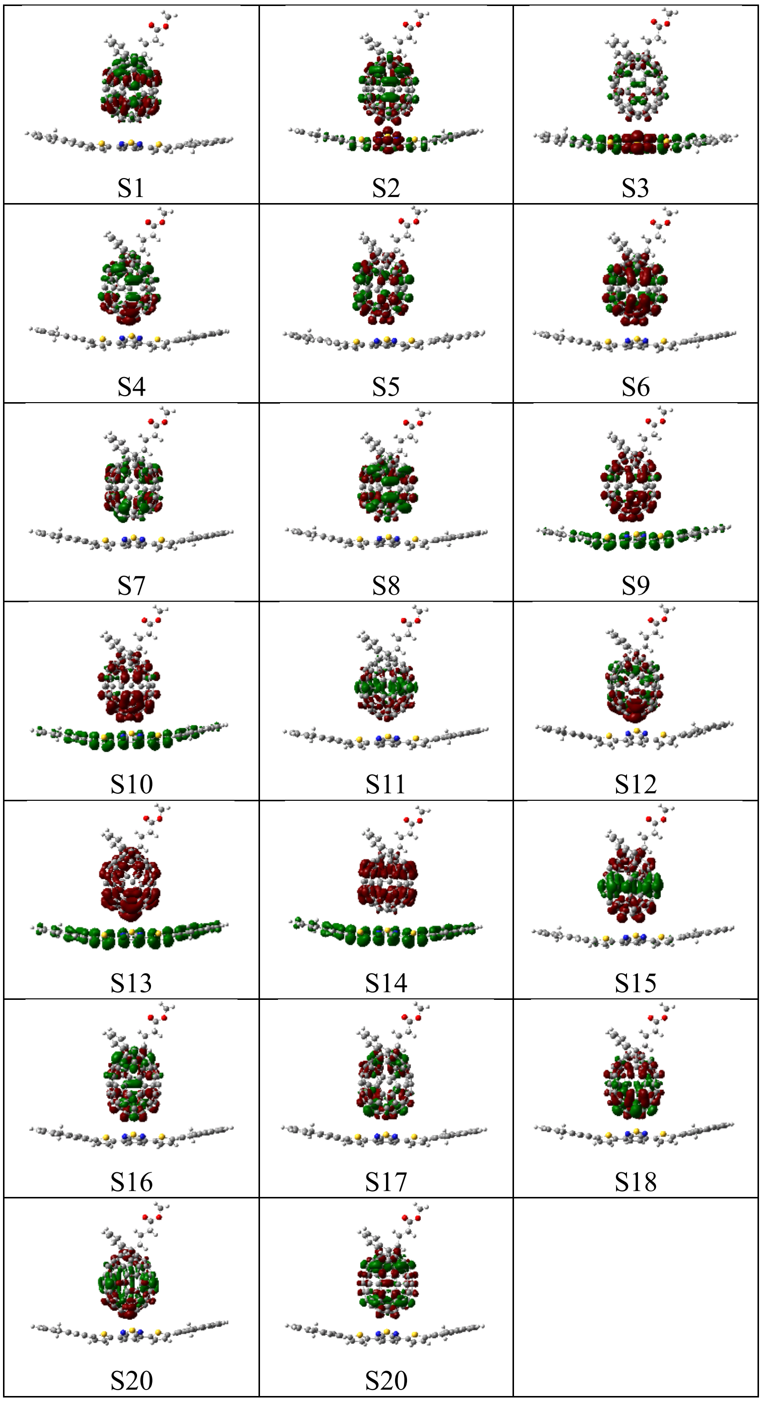

Figure S3. Charge difference density (CDD) of [C70] PCBM/APFO3, where the green and red stand for the hole and electron, respectively.
